# Aging Structure, Mechanical Properties, and ZnO Piezoelectric Coating-Based Ultrasonic Response of 15CrMo Steel

**DOI:** 10.3390/ma19020255

**Published:** 2026-01-08

**Authors:** Huayong Hu, Yanbing Zhang, Xiangdong Ma, Zhiping Fu, Jie Liu, Jun Zhang, Bing Yang

**Affiliations:** 1 Special Equipment Safety Supervision Inspection Institute of Jiangsu Province, Nanjing 210036, China; 2 School of Power & Mechanical Engineering, Wuhan University, Wuhan 430072, China

**Keywords:** 15CrMo steel, microstructure, mechanical properties, ZnO piezoelectric coating, ultrasonic response

## Abstract

The ZnO piezoelectric coatings were deposited on the surface of 15CrMo steels by magnetron sputtering to directly excite the ultrasonic signal, effectively solving the coupling problem between the traditional probe and the pipe surface. The microstructure, mechanical properties, and ultrasonic longitudinal wave velocity of the aged samples were carried out systematically. The spheroidization grade of pearlite, evolution of carbide morphology, hardness, strength, and ultrasonic wave velocity were systematically analyzed. As the degree of aging intensifies, the material undergoes significant pearlite spheroidization and carbide coarsening. The Vickers hardness drops from 158 HV in the original state to 134.2 HV, and the yield strength and tensile strength decrease by 22.7% and 17.9%, respectively. The ultrasonic longitudinal wave velocity shows a monotonically upward trend with the increase in spheroidization grade, increasing from 5925.6 m/s in the original state to 5976 m/s at the highest spheroidization grade.

## 1. Introduction

Power plant boiler pipes, serving as critical components of energy systems, have their safe operation directly determining the reliability of the entire power infrastructure [1,2]. Under extreme service conditions involving high temperatures, high pressures, and complex stress coupling, pipeline materials undergo irreversible microstructural evolution and performance degradation, posing potential hazards that threaten power plant safety [3,4]. Globally, over 30% of unplanned power plant outages are directly attributable to pipeline material aging, resulting in annual economic losses amounting to billions of dollars [5]. This severe situation underscores the urgency and importance of developing advanced aging assessment technologies.

Conventional aging assessment methods primarily rely on destructive techniques, such as metallographic analysis and mechanical property testing [6,7,8]. Although regarded as the “gold standard” for evaluating material aging states, their inherent limitations severely constrain real-time safety monitoring of in-service equipment. The destructive nature of sampling compromises pipeline structural integrity and introduces new safety risks. The discrete sampling approach fails to comprehensively reflect the overall aging state of the pipeline system [9]. Protracted testing cycles cause significant result delays, unable to meet the modern power system’s demand for real-time equipment condition monitoring. These deficiencies render traditional methods inadequate for providing timely and effective technical support for preventive maintenance decisions [10].

Ultrasonic testing technology demonstrates unique advantages in aging assessment due to its non-destructive nature, high penetration capability, and sensitivity to microstructural changes in materials [11,12]. However, the practical application of ultrasound at high temperatures presents formidable challenges, primarily centered on the transduction method. The existing ultrasonic testing methods can be classified into contact ultrasonic testing and non-contact ultrasonic testing. Electromagnetic acoustic transducers (EMATs), as a representative non-contact method, do not require couplant. Oh et al. [13] analyzed the degradation of the superheater tubes in thermal power plants through electromagnetic ultrasonic testing technology. However, their low signal-to-noise ratios, high cost, and limited operational temperature ceilings constrain their use in many industrial high-temperature scenarios. Conventional contact methods utilize piezoelectric probes coupled to the component via a couplant layer. Wang et al. [14] evaluated the aging behavior of thermal butt fusion joints in high-density polyethylene natural gas pipelines using contact ultrasonic technology. Vacuum grease gel was used for the contact between the transducers and samples to increase the ultrasonic wave signal quality. Oh et al. [15] evaluated the high-temperature degradation of platen superheater tubes in thermoelectric power plants by using contact surface ultrasonic measurement technology. Shen et al. [16] studied the ultrasonic signal of pearlite spheroidization damage in 15CrMo steel by contact ultrasonic testing. Glycerol was selected as the acoustic coupling agent due to its favorable characteristics of low acoustic attenuation and high viscosity. In high-temperature environments, this paradigm fails. Organic couplants degrade or evaporate, while specialized high-temperature couplants can be costly, difficult to apply consistently, and may only be effective for short-term measurements. An alternative contact approach involves bonding piezoelectric elements (PZT wafers or patches) using high-temperature adhesives. This method introduces an uncontrolled and thermally unstable adhesive layer, whose properties vary with temperature, compromising measurement accuracy and long-term reliability, and often leading to sensor failure through delamination. To circumvent these transduction barriers, the direct integration of piezoelectric materials onto the component surface as thin films has emerged as a promising technological pathway. This approach inherently eliminates the external couplant, promises better thermal and mechanical compatibility with the substrate, and enables the vision of permanent, embedded sensor networks [17,18,19].

In this study, we introduce a novel non-destructive testing methodology centered on the direct deposition of ZnO piezoelectric coatings onto 15CrMo steel substrates. This core innovation addresses the critical precision and coupling problem by creating an intimate, permanent piezoelectric interface. The ZnO piezoelectric coatings were deposited onto pipe surfaces to generate ultrasonic signals and enable real-time aging monitoring. Through systematic aging of 15CrMo steel under various temperatures and durations, we conducted detailed analyses of the microstructure, mechanical properties, and ultrasonic velocity of materials at different aging stages. The research determined the qualitative relationship between longitudinal wave velocity and spheroidization grade, providing a theoretical basis for evaluating the aging state of pipelines.

## 2. Experimental Details

### 2.1. Materials Preparation

The 15CrMo steels (Baosteel Group Co., Ltd., Shanghai, China) were subjected to normalizing treatment, featuring the initial microstructure of ferrite and lamellar pearlite. The chemical composition of 15CrMo was determined through experiments, and the results were presented in Table 1.

**Figure 1:**
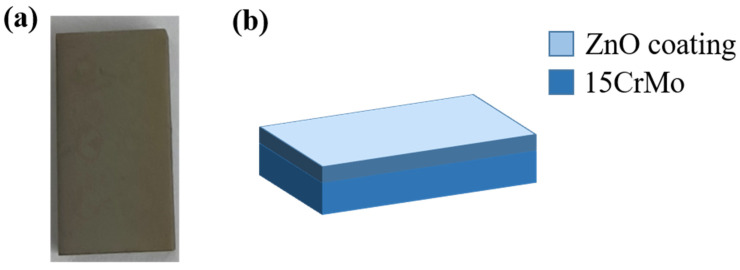
Macro picture (**a**) and schematic diagram (**b**) of the sample.

The aging temperature and time were selected according to the Larson–Miller formula, P = T(C + lgt) [20]. The C parameter of 15CrMo is 20.30, and the P parameter is P = 21,853. We chose temperatures of 610 °C, 630 °C, and 650 °C. The annealing times for each temperature are 100 h, 300 h, 1000 h, and 3000 h, respectively. After all the conditional annealing, we conducted microstructure observations on the samples and evaluated the degree of aging of the samples. Six samples of different aging grades were selected as representatives for testing. The corresponding sample designations were listed in Table 2. A macro picture and schematic diagram of the sample are shown in Figure 1. The length and width of the samples are 5 and 3 cm, respectively. The thickness of each sample was measured six times, and the average value was taken. The average thickness of samples with different degrees of aging is shown in Table 3. The ZnO piezoelectric coatings were deposited on the aged samples using a magnetron sputtering device (Shenyang LEBUY vacuum, V201, Shenyang, China) for ultrasonic testing. The 150 mm diameter pure ZnO target (99.999%) was used. The ratio of Ar to O_2_ is 1, and the chamber pressure is 2 Pa. The power of the RF power supply is 700 W, and the deposition time is 5 h.

### 2.2. Materials Characterization

The samples after aging were ground with sandpaper and polished with the polishing machine to form a bright and scratch-free mirror surface. Then, they were eroded with the 4% nitric acid alcohol solution, and the pearlite spheroidization structure was observed using an optical microscope (AxioVert.A1, ZEISS, Oberkochen, Germany). The surface morphology was investigated by scanning electron microscopy (SEM, TESCAN MIRA 3, TESCANBrno, Ltd., Oxford, UK). The Vickers hardness of samples was measured by the Vickers hardness tester (HVS-1000, Huayin Testing Instrument Co., Ltd., Yantai, China) using a regular tetrahedral diamond indenter with a load of 0.3 kg and a holding time of 10 s. Six points were taken from each sample to calculate the average values. The raw material of 15CrMo steel and the material with different degrees of spheroidization after aging treatment were processed into standard tensile specimens, and tensile tests were conducted using the tensile testing machine (MTS880, MTS Co., Ltd., Eden Prairie, MN, USA) with a tensile rate of 0.375 mm/min. Three sets of data were taken from each sample to calculate the average values. A DPR300 ultrasonic pulse receiver (Imaginant, New York, NY, USA) was used to collect ultrasonic signals excited by ZnO piezoelectric thin films. The gain of signal acquisition was 40 dB, and the voltage applied was 200 V.

## 3. Results and Discussion

### 3.1. Microstructure

**Figure 2:**
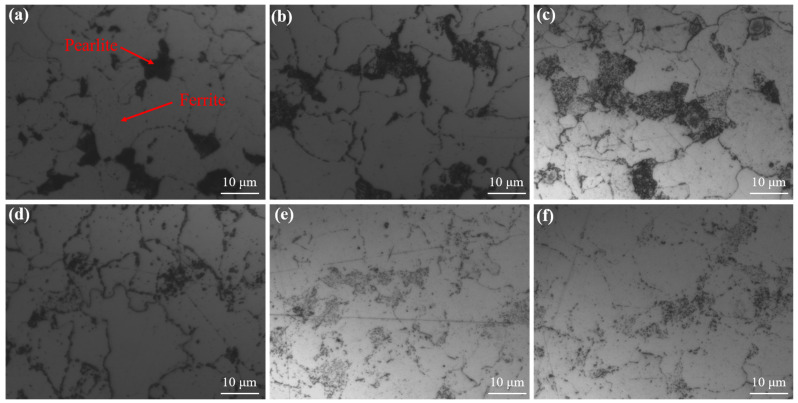
The microstructure of 15CrMo steels treated under different aging conditions. (**a**) 25 °C 0 h, (**b**) 630 °C 100 h, (**c**) 610 °C 1000 h, (**d**) 650 °C 300 h, (**e**) 630 °C 3000 h, (**f**) 650 °C 1000 h.

The spheroidization standards for 15CrMo steel pearlite used in thermal power plants are shown in Table 4. Figure 2 illustrates the evolution of the metallographic structure of specimens subjected to different temperatures and aging durations. Thermodynamically, the final products of austenite eutectoid decomposition should ideally be equilibrium α-ferrite and graphite carbon. However, even under slow cooling rates, the reaction typically yields α-ferrite and cementite, forming a lamellar pearlite structure [21]. The original 15CrMo steel sample, without high-temperature aging (Figure 2a), exhibits distinct lamellar carbides (M_3_C) within pearlite colonies, characteristic of a typical ferrite and pearlite microstructure, corresponding to Spheroidization Grade 1. The interlamellar spacing within pearlite colonies is clearly discernible, forming the basis for the material’s excellent strength [22]. Sample 2 (Figure 2b) shows largely intact pearlite regions with incipient discontinuity in lamellar features; cementite begins to fragment and spheroidize, and carbide particles appear at grain boundaries, corresponding to Grade 2. Sample 3 (Figure 2c) displays relatively complete pearlite regions with numerous spheroidized carbides, yet retains partial lamellar traces and increased grain boundary carbides, corresponding to Grade 3. Sample 4 (Figure 2d) shows essentially vanished pearlite regions, though their morphology is retained; the lamellar structure completely disappears, carbides fully spheroidize, and grain boundary carbides coalesce into chain-like structures, corresponding to Grade 4. Samples 5 and 6 (Figure 2e,f) exhibit the complete disappearance of pearlite, transitioning entirely to a spheroidized structure comprising coarse granular carbides distributed within the ferrite matrix, with significant carbide coarsening and grain boundary aggregation, corresponding to Grade 5. Increasing temperature and aging time initiate the fragmentation and spheroidization of lamellar cementite (Grades 2–3), dispersion of carbides within pearlite colonies, followed by gradual diffusion and aggregation at grain boundaries until the pearlite morphology vanishes, ultimately evolving into a fully spheroidized structure with coarse carbide particles on the ferrite matrix (Grades 4–5). Carbide aggregation at grain boundaries leads to boundary coarsening. For equal volume, lamellar structures possess greater surface area than spheroidal ones, resulting in higher surface energy. The second law of thermodynamics dictates that higher energy states tend spontaneously towards lower energy states. Thus, lamellar pearlite is considered a metastable structure, with an inherent tendency for cementite to transform into spheroidal particles and coarsen, a diffusion-based process [23]. Elevated temperatures accelerate atomic diffusion, facilitating the transformation of lamellar cementite into granular form and subsequent aggregation into larger spheroids, making pearlite spheroidization an inevitable microstructural evolution during prolonged high-temperature service [24,25]. Observation of variously aged specimens clearly reveals the progressive transition from metastable lamellar pearlite to spheroidized microstructure. With increasing holding time and temperature, spheroidization in 15CrMo steel intensifies: original lamellar pearlite gradually decomposes, pearlite regions become less distinct, grain boundary carbide quantity increases, and at Grade 5, pearlite region characteristics essentially disappear, with most carbides aggregated at grain boundaries and significantly coarsened.

**Figure 3:**
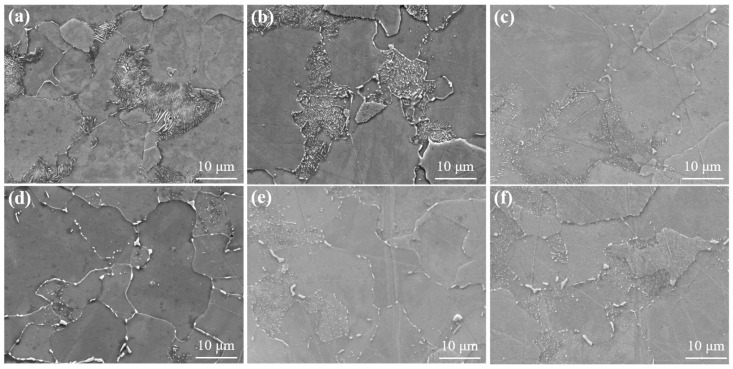
SEM micrographs of 15CrMo steel treated under different aging conditions. (**a**) 25 °C 0 h, (**b**) 630 °C 100 h, (**c**) 610 °C 1000 h, (**d**) 650 °C 300 h, (**e**) 630 °C 3000 h, (**f**) 650 °C 1000 h.

To more accurately evaluate the aging degree of 15CrMo steel, SEM analysis was performed on samples exhibiting varying degrees of spheroidization. The results are presented in Figure 3. In the original sample, the ferrite matrix contains lamellar pearlite (Figure 3a), which exhibits a high interfacial energy density. The continuous and extensive cementite/ferrite phase interfaces maintain the system in a pronounced thermodynamic metastable state. Regions with smaller radii of curvature—such as lamellar edges and defect sites—possess higher chemical potential, and the resulting chemical potential gradient serves as the primary driving force for microstructural evolution. Under elevated temperature conditions, the system reduces its total interfacial energy to achieve a lower energy state, which fundamentally drives the spontaneous spheroidization process [26]. With progressive aging, lamellar structures initially fracture at defect locations, forming a beaded morphology that evolves into discrete, nearly spherical carbide particles. Eventually, the carbides become significantly coarsened and non-uniformly distributed, with some accumulating at grain boundaries—a finding consistent with optical metallographic observations. The extent of sample aging reflects the evolutionary stages of pearlite spheroidization. Under thermal activation, carbon atoms migrate directionally via bulk and interfacial diffusion, leading to periodic necking of cementite lamellae at regions of non-uniform thickness or crystal defects (Figure 3b). This increases the interfacial curvature gradient and facilitates subsequent microstructural transformation. Notably, lamellar fragmentation does not occur uniformly but exhibits marked asynchrony among different pearlite colonies, which is attributed to variations in initial lamellar spacing, crystallographic orientation, and local defect density. The fragmented cementite gradually transforms from irregular morphologies into near-equilibrium spherical particles through the classical Ostwald ripening mechanism (Figure 2c,d). Due to higher solubility at sharp corners with small radii of curvature, carbon atoms dissolve and diffuse through the ferrite matrix to deposit on surfaces with larger radii of curvature. The transformation rate of short rod-like fragments into spherical particles is considerably faster than the lamellar fragmentation process. As aging progresses (Figure 2e,f), the complete dissolution of pearlite colonies signifies the reconstruction of the original microstructure. Carbides undergo significant spatial redistribution, transitioning from a relatively uniform dispersion to preferential segregation at grain boundaries. Grain boundaries, acting as high-diffusivity pathways, provide efficient channels for long-range carbon transport. According to grain boundary diffusion theory, the diffusion coefficient of carbon atoms along grain boundaries can be 3 to 6 orders of magnitude greater than that of bulk diffusion, enabling carbide accumulation in grain boundary regions via a “dissolution–diffusion–reprecipitation” mechanism [27]. A significant lattice mismatch exists between the carbide and the ferrite matrix, generating coherent strain energy. Grain boundary regions can partially accommodate and relieve this strain energy, thereby offering energetically favorable sites for carbide nucleation and growth. Enhanced carbide coarsening is observed at triple junctions (Figure 3f), where the overlapping diffusion fields of three adjacent grains create a convergent diffusion effect, accelerating carbide aggregation and growth.

**Figure 4:**
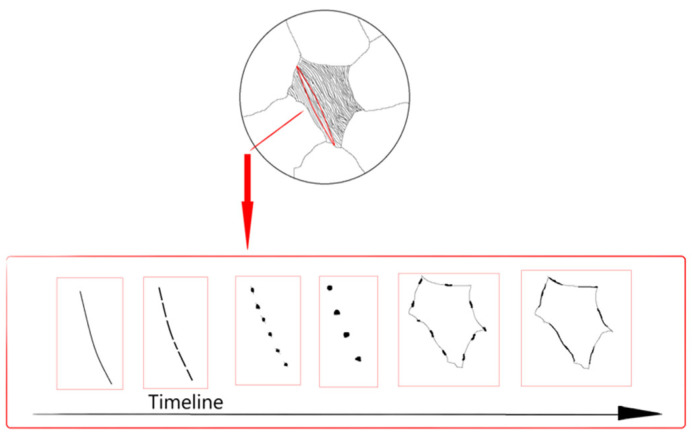
Schematic of lamellar pearlite evolution during high-temperature aging.

The evolution from lamellar to spheroidal pearlite during high-temperature aging is schematized in Figure 4. With progressing aging, intact lamellar carbides transform into a mixture of short rod-like and dot-like carbides. Subsequently, the proportion of rod-like carbides decreases, transitioning to dot-like carbides, which aggregate, coarsen, and diffuse towards grain boundaries, forming chain-like carbides. These grain boundary chains further grow and merge with adjacent chains, leading to grain boundary coarsening. Carbide accumulation also occurs at triple junctions, weakening boundary strength and facilitating crack initiation, severely impacting material properties. Lamellar pearlite’s high interfacial energy renders it thermodynamically unstable. High temperature provides the driving force for atomic diffusion, enabling cementite lamellae to reduce total interfacial energy via “fragmentation-spheroidization”, causing material aging and performance degradation.

### 3.2. Mechanical Properties

**Figure 5:**
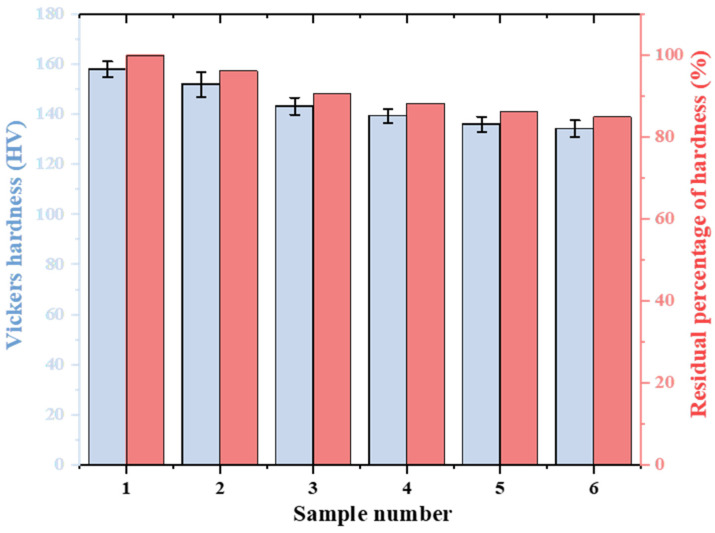
Vickers hardness and residual hardness percentage of 15CrMo steel at different spheroidization grades.

Vickers hardness and residual hardness percentage for samples aged under different conditions are shown in Figure 5. The Vickers hardness monotonically decreases with increasing the degree of aging, dropping from the original 158 HV to 134.2 HV. The high interfacial energy of lamellar pearlite makes it thermodynamically unstable [25]. High temperature provides the driving force for atomic diffusion, causing the cementite laminate to reduce the total interfacial energy through spheroidization [28]. During the spheroidization process, solute atoms in the ferrite matrix diffuse through vacancies and grain boundaries, forming carbides near the grain boundaries [29]. As spheroidization proceeds, the aggregation of carbides at the grain boundaries depletes solute atoms, weakening solid solution strengthening and reducing the microhardness of ferrite [30]. In practical applications, the correlation between pearlite spheroidization grade and microhardness can be quickly and simply evaluated through sampling and hardness to assess the material condition and aging degree.

**Figure 6:**
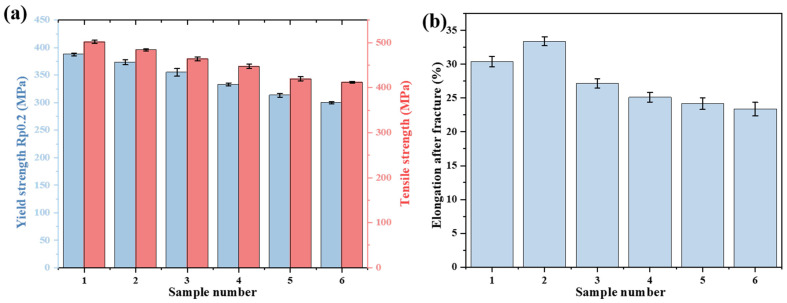
(**a**) Yield strength and ultimate tensile strength and (**b**) post-elongation of 15CrMo steel at different spheroidization grades.

Tensile test results for yield strength (YS) and ultimate tensile strength (UTS) of aged samples are shown in Figure 6a. From unspheroidized to fully spheroidized states, UTS decreases from 501.5 MPa to 411.9 MPa, and YS decreases from 387.7 MPa to 299.8 MPa, representing reductions of 17.9% and 22.7%, respectively. The decline in strength correlates with the disruption of the continuous lamellar carbide structure. This lamellar structure acts as a formidable barrier to dislocation motion [31]. Its fragmentation and spheroidization drastically reduce the effective obstacle area, enabling dislocations to bypass the now-spheroidal particles via the Orowan mechanism at a lower applied stress [32].

Post-elongation for different aging degrees is shown in Figure 6b. Elongation initially increases to 33.3% in moderately aged samples (up to Grade 3) before decreasing sharply to 23.5% in fully spheroidized samples (Grades 4–5). In the early stages of spheroidization, the replacement of sharp, stress-concentrating lamellae with rounded particles delays microcrack initiation, allowing for greater uniform plastic deformation. Simultaneously, the depletion of solute atoms from the ferrite matrix due to carbide formation softens the matrix, facilitating dislocation glide and enhancing plasticity. However, in advanced aging stages, our microstructural evidence points to a different dominant mechanism. The coarse, chain-like carbides at grain boundaries (Figure 3e,f) become potent sites for microcrack nucleation and provide easy paths for intergranular crack propagation. This transition from a ductile, transgranular fracture mode to a brittle, intergranular one is the primary reason for the eventual loss of toughness in severely aged materials [33].

### 3.3. Ultrasonic Response

**Figure 7:**
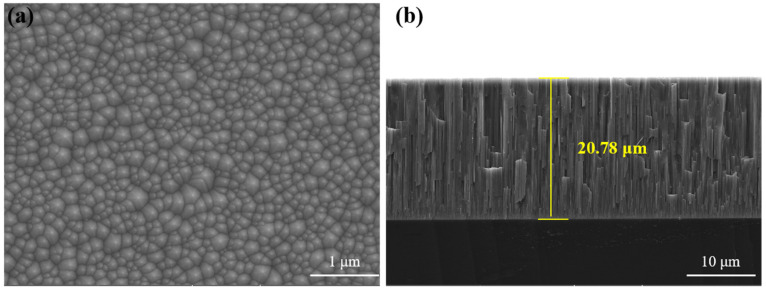
The surface (**a**) and cross-sectional (**b**) morphologies of the ZnO coatings.

**Figure 8:**
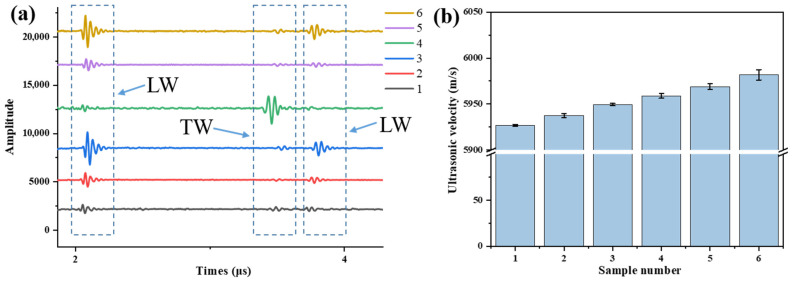
(**a**) Ultrasonic pulse–echo response and (**b**) ultrasonic wave velocity of 15CrMo steel at different spheroidization grades.

The surface and cross-sectional morphologies of the ZnO coatings are shown in Figure 7. The surface of the coating was closely arranged. The thickness of the ZnO piezoelectric coating was 20.78 μm. The cross-section of the coating showed columnar crystal growth, with a dense structure and no obvious defects. For rapid, convenient, non-destructive assessment of material aging, ultrasonic testing was performed on samples with different spheroidization grades. The ultrasonic pulse-echo response is shown in Figure 8a. The material generates three back-wall echoes: two longitudinal waves (LW) and one transverse wave (TW), with successively decreasing amplitude, which indicates that the direct deposition of ZnO piezoelectric coatings can effectively solve the ultrasonic coupling problem and meet the requirements of long-term applications. Compared with the ultrasonic testing of piezoelectric wafers [34] it has higher precision, is less likely to fall off, and is suitable for higher temperatures. Longitudinal waves were selected for velocity calculation based on the sample thickness and the ultrasonic echo time, as shown in Figure 8b. The time accuracy of our ultrasonic testing equipment is 0.1 ns. The calculated error of the ultrasonic velocity equipment is less than 4 m/s. Multiple signal acquisitions were conducted for each sample to calculate the average ultrasonic velocity. The thickness of the samples was measured multiple times, and the average value was calculated to ensure the reduction in errors and the repeatability of the experiment. Ultrasonic velocity increases monotonically with material aging. The longitudinal wave velocity in the solid is given by [35]:

(1) $$v=\sqrt{\frac{E(1-\nu )}{\rho (1+\nu )(1-2\nu )}}$$

where E is Young’s modulus, ρ is density, and ν is Poisson’s ratio. During aging, density and Poisson’s ratio remain virtually unchanged; thus, the velocity increase primarily reflects an increase in Young’s modulus E. In the original 15CrMo steel, alloying elements (Cr, Mo, C) are in solid solution or in metastable cementite. During the aging process, the C element precipitated and aggregated towards the grain boundaries to form carbide particles, which have been observed in the SEM images. These precipitates are coherent or semi-coherent with the ferrite matrix. The associated coherency strain fields effectively increase the local lattice stiffness. From a phonon propagation perspective, sound waves travel faster through these “pre-stressed” regions, leading to an overall increase in the measured ultrasonic velocity [35]. The original material contains a high density of randomly distributed dislocations and residual stresses, which act as scattering centers for ultrasonic waves, reducing velocity [36]. After aging treatment, the precipitation of fine, stable alloy carbides and the rearrangement and recovery of dislocations from their initial, high-density state reduce the scattering of ultrasonic waves and enhance the lattice’s effective stiffness, resulting in an increase in ultrasonic velocity. As the degree of aging deepens, the ultrasonic velocity increases, revealing a correlation between them. The results show that the direct deposition of ZnO piezoelectric coatings can qualitatively analyze the aging degree of 15CrMo boiler tubes through ultrasonic velocity measurement.

## 4. Conclusions

The microstructure, mechanical properties, and ultrasonic longitudinal wave velocity of 15CrMo steels with different degrees of aging were systematically studied. High-temperature aging drives significant pearlite spheroidization and carbide coarsening. Carbides gradually change from continuous layered to isolated spherical, accompanied by obvious grain boundary aggregation. With the deepening of aging, the Vickers hardness decreased from 158 HV to 134.2 HV, and the yield strength and tensile strength decreased by 22.7% and 17.9%, respectively. The ultrasonic velocity increases monotonically with the increase in spheroidization degree, from 5925.6 m/s in the initial state to 5976 m/s, which is in sync with the degradation trend of mechanical properties. The feasibility of evaluating the aging state of 15CrMo steel by directly depositing ZnO piezoelectric coatings using ultrasonic wave velocity was verified. In the future, we will conduct more detailed experiments to quantitatively assess material aging and apply it to more materials.

## Figures and Tables

**Table 1:** The chemical composition (Mass Fraction, %) of 15CrMo steels.

Element	C	Si	Mn	P	S	Cr	Mo
Content	0.16	0.27	0.58	0.032	0.027	0.89	0.36

**Table 2:** The aging treatment parameters corresponding to the sample number.

Sample Number	1	2	3	4	5	6
Aging parameters	25 °C 0 h	630 °C 100 h	610 °C 1000 h	650 °C 300 h	630 °C 3000 h	650 °C 1000 h

**Table 3:** The average thickness of samples with different degrees of aging.

Sample Number	1	2	3	4	5	6
Thickness/mm	4.949	5.012	5.083	4.956	5.046	5.046

**Table 4:** Microstructure characteristics of pearlite spheroidization of the15CrMo steel [23].

Spheroidization Degree	Spheroidization Grade	Organizational Characteristics
Unspheroidized (Original state)	Level 1	The characteristics of pearlite are clear, and the carbides in pearlite are in lamellar form
Tendency to spheroidize	Level 2	The pearlite region is basically intact, and the lamellar carbides tend to be divided/scattered, with a few carbides at the grain boundaries
Mild spheroidization	Level 3	The pearlite region is relatively complete; some carbides are granular, and the number of carbides at the grain boundaries has increased
Moderate spheroidization	Level 4	The pearlite region still retains its morphology. Most of the carbides in pearlite are granular with a high degree of dispersion, and the carbides at the grain boundaries appear in a chain-like pattern
Complete spheroidization	Level 5	The pearlite morphology has vanished, leaving only a small amount of granular carbides distributed at the grain boundaries, with a significant increase in particle size

## Data Availability

The original contributions presented in this study are included in the article. Further inquiries can be directed to the corresponding authors.
